# Astragaloside IV Inhibits Galactose-Deficient IgA1 Secretion *via* miR-98-5p in Pediatric IgA Nephropathy

**DOI:** 10.3389/fphar.2021.658236

**Published:** 2021-04-16

**Authors:** Caiqiong Liu, Xiaoyan Li, Lanjun Shuai, Xiqiang Dang, Fangrong Peng, Mingyi Zhao, Shiqiu Xiong, Ying Liu, Qingnan He

**Affiliations:** ^1^ Department of Pediatrics, The Second Xiangya Hospital, Central South University, Changsha, China; ^2^ Department of Pediatrics Nephrology, Children’s Medical Center, The Second Xiangya Hospital, Central South University, Changsha, China; ^3^ Department of Pediatrics, The Third Xiangya Hospital, Central South University, Changsha, China

**Keywords:** immunity, galactose-deficient IgA1, astragaloside IV, miR-98-5p, IgA nephropathy, *β*-1, 3-galactosyltransferase

## Abstract

**Purpose:** The factor associated with IgA nephropathy (IgAN) is an abnormality of IgA known as galactose-deficient IgA1 (Gd-IgA1). The purpose of this study was to determine the molecular role played by miRNAs in the formation of Gd-IgA1 in IgAN and investigate the regulatory role of Astragaloside IV (AS-IV) in miRNAs.

**Patients and methods:** Bioinformatics analysis, along with functional and mechanistic experiments, were used to investigate the relationship and function of miRNA, β-1, 3-galactosyltransferase (C1GALT1), Gd-IgA1, and AS-IV. Analyses involved a series of tools, including quantitative real-time polymerase chain reaction (qRT-qPCR), Western blot, enzyme-linked immunosorbent assay (ELISA), Vicia Villosa lectin-binding assay (VVA), Cell counting kit-8 assay (CCK-8), and the dual-luciferase reporter assay.

**Results:** miRNA screening and validation showed that miR-98-5p was significantly upregulated in the peripheral blood mononuclear cells (PBMCs) of pediatric patients with IgAN compared with patients diagnosed with mesangial proliferative glomerulonephritis (MsPGN) and immunoglobulin A vasculitis nephritis (IgAV-N), and healthy controls (*p* < 0.05). Experiments with the dual-luciferase reporter confirmed that miR-98-5p might target C1GALT1. The overexpression of miR-98-5p in DAKIKI cells decreased both the mRNA and protein levels of C1GALT1 and increased the levels of Gd-IgA1 levels; these effects were reversed by co-transfection with the C1GALT1 plasmid, and *vice versa*. In addition, AS-IV downregulated the levels of Gd-IgA1 level in DAKIKI cells by inhibiting miR-98-5p.

**Conclusions:** Our results revealed that AS-IV could inhibit Gd-IgA1 secretion *via* miR-98-5p. Increased levels of miR-98-5p in pediatric IgAN patients might affect the glycosylation of IgA1 by targeting C1GALT1. In addition, our analyses suggest that the pathogenesis of IgAN may differ from that of IgAV-N. Collectively, these results provide significant insight into the pathogenesis of IgAN and identify a potential therapeutic target.

## Introduction

IgA nephropathy (IgAN) is the most prevalent form of primary glomerulonephritis and predominantly affects children and young adults. The most striking feature of IgAN is the mesangial deposition of IgA or IgA-containing immune complexes (Cambier et al., 2020; Coppo and Robert, 2020). IgAN may represent a systemic immune dysregulation rather than an intrinsic abnormality of resident renal cells (Moroni et al., 2019). The mainstream pathogenesis of IgAN is described by the multi-hit hypothesis which involves four major steps: the overproduction of galactose-deficient IgA1 (Gd-IgA1) and autoantibodies against Gd-IgA1, the formation of circulating immune complexes (CICs) which are subsequently deposited in the glomeruli, and the activation of mesangial cells, thus resulting in renal injury (Suzuki et al., 2011). These data indicate that Gd-IgA1 plays a crucial role in the pathogenesis of IgAN. However, we know very little about the molecular mechanisms underlying these processes.

Human IgA has two subclasses: IgA1 and IgA2 (Placzek et al., 2018). The IgA1 molecule differs from the IgA2 subclass in that it features a unique hinge region. This region has many serine threonine and proline residues, and nine potential O-glycosylation sites (usually, 3–6 sites of each hinge region are O-glycosylated); these residues can be affected by various disorders (Novak et al., 2018). IgA1 O-glycosylation needs N-acetyl-galactosamine (GalNAc) to be added to the serine or threonine residues, followed by galactose (Gal). β-1,3-galactosyltransferase (C1GALT1) catalyzes the addition of Gal residue. Finally, O-glycosylation is completed by the addition of sialic acid residues (Lai et al., 2019). Gd-IgA1 can then be formed as the result of the reduced activity or expression of C1GALT1, and the elevation of α2,6-sialyltransferase (ST6GALNAcII) (Lai et al., 2019). Genome-wide association analyses have revealed the crucial role of the C1GALT1 in the secretion of Gd-IgA1 (Kiryluk et al., 2017). However, the C1GALT1 genotype explains only 3% of the variance in Gd-IgA1 levels, thus suggesting that transcriptional or post-transcriptional regulation also plays a key role (Kiryluk et al., 2017).

MicroRNAs (miRNAs) are endogenous small non-coding RNAs that can down regulate targeted mRNAs by pairing to specific sites within the 3’-untranslated regions (3’UTRs) (Wang J. et al., 2019). Previous research suggests that miRNAs target up to 60% of human mRNAs (Hennino et al., 2016). Recent insights have also revealed that miRNAs regulate a variety of cellular processes, including immune reactions and the pathogenesis of kidney disease (Zhao et al., 2019). miRNAs are also closely related to immune responses. For instance, the upregulation of miR-210 has been shown to inhibit B cell activity and autoantibody synthesis, while the upregulation of miR-148a and miR-19-92 disrupted the central tolerance of B cells and accelerated autoimmunity (Mok et al., 2013; Gonzalez-Martin et al., 2016; Xiao et al., 2020). This suggests that miRNAs play an important role in renal diseases.

*Astragalus membranaceus* is a traditional Chinese herb that is often used in the clinical treatment of kidney diseases (Wang E. et al., 2020). Astragaloside IV(AS-IV) is a saponin molecule isolated from *A. membranaceus*. AS-IV exhibits a range of beneficial biological and pharmacological activities, including anti-inflammatory effects, immunomodulatory effects, cardioprotection, and renoprotection (Wang E. et al., 2020; Wang Z. et al., 2020). Recent studies have reported that AS-IV can suppress kidney fibrosis and exert beneficial effects on diabetes and its related complications *via* multiple mechanisms (Wang E. et al., 2020). AS-IV is also known to function as a regulator of miRNAs levels (Gong et al., 2018; Cui et al., 2020). We therefore hypothesized that miR-98-5p might participate in the pathological process of Gd-IgA1 production and could be regulated by AS-IV in IgAN.

## Methods

### Bioinformatics Analysis of miRNAs

First, we used specific keywords (“IgA nephropathy, IgA nephritis, or Berger’s disease” and “peripheral blood mononuclear cells (PBMCs)” and “microRNA”) to search the Gene Expression Omnibus (GEO) (http://www.ncbi.nlm.nih.gov/geo/); this allowed us to acquire the GSE25590 miRNA microarray dataset (Serino et al., 2012). Next, we downloaded the raw data and used the normalizeBetweenArrays function in the Limma package of R to normalize the expression values within the quartile and then obtained standard expressions by Log_2_ transformation. Differentially expressed miRNAs (DEmiRNAs) were screened using the Limma package of R using |log_2_fold change (FC)| > 1 and *p*-value < 0.05 as thresholds. Next, we used TargetScan (http://www.targetscan.org/vert_72/) and miRDB (http://mirdb.org/) to predict the upstream regulated miRNAs for C1GALT1. miRNAs that were common to both databases were defined as predicted miRNAs. The intersection between the DEmiRNAs and the predicted miRNAs were then used for further analysis.

### The Collection of Samples From Patients and Controls

A total of 24 pediatric cases were enrolled in our study; these cases were divided into four groups with six cases in each group: a group of patients with IgAN, a group of patients with mesangial proliferative glomerulonephritis (MsPGN), a group of patients with immunoglobulin A vasculitis nephritis (IgAV-N), and a group of healthy controls. All patients had normal renal function. None of the patients underwent treatment with steroids, immunosuppressive agents, antibiotics, or non-steroidal anti-inflammatory agents. All healthy controls had a normal urine test. We collated clinical data from each patient and collected venous blood on the day of kidney biopsy (for patients) or recruitment (for healthy controls). Blood samples were treated with an anticoagulant and centrifuged at 1000 g at room temperature for 10 min; we then collected the supernatant (plasma) for analysis. The remaining blood was diluted with an equal volume of Phosphate Buffer Saline (PBS). Next, we used a Ficoll-Hypaque (Ficoll-Paque Plus, Solarbio, Beijing) gradient (800 g for 30 min at room temperature) to isolate PBMCs *via* density separation. The layer of PBMCs was collected and washed three times with PBS. Both PBMCs suspension and plasma were stored at −80°C for subsequent experiments. This study was approved by the Medical Ethical Committee of the Second Xiangya Hospital of Central South University (No. 2019–190S (161), Changsha, China). Written informed consent was signed by every case’s legal guardian or next of kin.

### Cell Culture, Transfection, and Treatment

The DAKIKI cell line was a gift from Professor Liu Youxia of Tianjin Medical University General Hospital. This cell line was authenticated by short tandem repeat profiling (Genetic Testing Biotechnology, Suzhou, China). DAKIKI cells were cultured in RPMI 1640 medium containing 10% heat-inactivated fetal bovine serum (FBS), 1% penicillin-streptomycin (Shanghai, China); this was carried out in a humidified environment of 95% atmospheric air and 5% CO_2_. miR-98-5p mimics and inhibitors (anti-miR-98-5p oligonucleotides) (Krützfeldt et al., 2005), silent C1GALT1 (si-C1GALT1) and silent scramble (si-scramble), pc-DNA-C1GALT1 and pc-DNA-vector plasmids, were purchased from Jikai Genechem Co., Ltd. (Shanghai China). The sequences of the si-C1GALT1 and si-scramble were as follow: si-C1GALT1, 5’-TAT​ACG​TTC​AGG​TAA​GGT​AGG-3’ and si-scramble, 5’-TTC​TCC​GAA​CGT​GTC​ACG​T-3’. Transfection was performed using Mirus TransIT-TKO transfection reagent (Mirus Inc., Madison, WI, United States) in accordance with the manufacturer’s guidelines. Mock transfection was carried out with a mock reagent. DAKIKI cells were treated with AS-IV (Yuanye biomart, Shanghai, China) at different concentrations (0, 5, 10, 20, 40, and 80 μM) for 24 h. Then, the DAKIKI cells were treated with either vehicle or AS-IV (20 μM) and transfected with miRNA mimics.

### Quantitative Real-Time Polymerase Chain Reaction

Total RNA was extracted from isolated PBMCs and DAKIKI cells with TRIzol Reagent (Invitrogen, CA, United States). Reverse Transcription Systems (for miRNA: CW2141, for mRNA: CW2569, CoWin Biosciences, Beijing, China) were used to reverse transcribe 1 μg of total RNA into cDNA. Levels of miR-98-5p, miR-152-3p, and C1GALT1, were then determined by qRT-PCR using the UltraSYBR Mixture (CW2601, CoWin Biosciences, Beijing, China) on a qRT-PCR detection system (Thermo Fisher, Pikoreal96, United States). Primers are shown in Supplementary Table S1. U6 snRNA and β-actin were used for normalization. Gene expression levels were calculated by the 2^−ΔΔCT^ method. All experiments were replicated three times.

### Western Blotting

Total protein was extracted from isolated PBMCs and cultured cells using a protein extraction kit (Beyotime, Shanghai, China). Protein concentrations were determined using the BCA Protein Assay Kit (CW 2011, CoWin Biosciences, Beijing, China). The proteins were then separated by 10% sodium dodecyl sulfate polyacrylamide gel electrophoresis (SDS-PAGE) and transferred to nitrocellulose membranes (Pall Corporation, United States). Next, the membranes were blocked in 5% skimmed milk at room temperature for 2 h and probed at 4°C overnight with anti-C1GALT1 (1: 450; rabbit-anti-human; Santa Cruz Biotechnology, TX, United States), followed by incubation with a HRP-conjugated goat-anti-rabbit IgG antibody (1:7,000, goat-anti-rabbit, Proteinte, Chicago, United States) at room temperature for 2 h. Bands were then detected by an electrochemiluminescence detection kit (Advansta, California, United States) to produce a chemiluminescence signal that was captured on X-ray film. The same membranes were stripped and then re-probed with an anti-β-actin antibody (1:5,000 mouse-anti-human, Proteintech, Chicago, United States). This was then followed by incubation with a HRP-conjugated goat anti-mouse IgG antibody (1:5,000, goat-anti-mouse; Proteintech, Chicago, United States). Bands were then detected and captured, as described previously.

### IgA1 and Galactose-Deficient IgA1 Measurement

Levels of total IgA1 levels in plasma or the supernatant of DAKIKI cell were determined in duplicate by ELISA, in accordance with the manufacturer’s recommendations. In brief, 96-well immunoplates were coated with mouse anti-human IgA1 antibody (SouthernBiotech, United States) at 4°C overnight. After blocking with BSA, samples or standard human IgA1 (Santa Cruz Biotechnology, United States) were incubated (in duplicate) at 37°C for 2 h. Mouse biotin-labeled anti-human IgA1 specific antibodies (Southern Biotech Associates) were added and incubated for 1 h, then incubated with HRP-labeled Streptavidin (Beyotime, Shanghai, China) for 1 h. Positive staining was developed with tetramethyl benzidine dilution (TMB) and detected at 450 nm. The concentration of IgA1 was then determined by the use of a standard curve.

Gd-IgA1 levels were measured by the Vicia Villosa lectin-binding assay (VV, Vector Laboratories Associates, Peterborough, United Kingdom) (VVA). Immunoplates were coated with anti-IgA1 antibody and blocked with BSA, as described earlier. Next, 100 μl per well with 100 ng IgA1 of each sample (according to the concentration of IgA1) were added in duplicate. The plates were incubated at 4°C overnight, then incubated with biotinylated VV lectin (Vector Laboratories, Peterborough, United Kingdom) at 37°C for 2 h. Plates were then incubated with HRP-labeled Streptavidin (Beyotime, Shanghai, China) for a further 1 h. Positive staining was then developed and detected as described above; optical density (OD) units for each sample were expressed relative to IgA1(%VV).

### Cell Counting Kit-8 Assay

The CCK-8 toxicity assay was used to analyze the potentially toxic effects of AS-IV on the viability of DAKIKI cells. In brief, 4 × 10^5^ cells per well were seeded into 96-well plates and treated with different concentrations of AS-IV (0, 5, 10, 20, 40, and 80 μm) for 24 h. Then, 20 μl of Cell Counting Kit-8 solution (CCK-8, Dojindo Molecular Technologies, Kyushu, Japan) was added into each well. After incubation at 37°C for 4 h, the OD of each well was analyzed using a Microplate Reader (MB-530, HEALES, Shenzhen, China) at 450 nm.

### Dual-Luciferase Reporter Assay

The dual-luciferase reporter assay is a reporting system for detecting firefly luciferase activity using luciferin as a substrate. Luciferase can catalyze the oxidation of Luciferin to oxyluciferin. Bioluminescence is emitted during the oxidation of Luciferin; this bioluminescence can be measured with a fluorescence analyzer. The dual luciferase assay involves firefly luciferase and ranilla luciferase. One of these enzymes is used as an internal reference while the other is attached to the target gene 3’-UTR; this can be inhibited by miRNA and can therefore be used for target gene validation.

The potential complementary binding sequence between C1GALT1 and miR-98-5p was predicted by TargetScan and miRDB. Reporter constructs containing the wild-type (WT) or mutant-type (MUT) human C1GALT1 3’-UTRs synthesized by HonorGene (Changsha, China), were cloned into the pHG-MirTarget-C1GALT1 luciferase reporter vector (HonorGene, Changsha, China). Then, 293 A cells were seeded in 24-well plates and co-transfected with 2 μg pHG-MirTarget-C1GALT1-3U-WT or pHG-MirTarget-C1GALT1-3U-MUT, and 50 nm miR-98-5p mimic, or mimic NC by Lipofectamine 2000 reagent (Invitrogen; Thermo Fisher Scientific, Inc.). Firefly and Renilla luciferase activities were then determined by dual-luciferase reporter assays (Promega, GloMax 20/20 United States) at 48 h post-transfection in accordance with the manufacturer’s instruction. Values were normalized with Renilla luciferase.

### Statistical Analysis

Statistical analyses were performed with IBM-SPSS version 22.0 (Chicago, United States) statistical software and graphs were plotted with GraphPad Prism 7.0 (San Diego, CA). For continuous variables, normally distributed data were expressed as mean ± SD and were compared by an independent-sample t-test or one-way analysis of variance (ANOVA); Pearson’s correlation was also used if necessary. Other forms of data were expressed as the median (first quartile and third quartile) and analyzed by the Mann-Whitney U test. A two-tailed *p* < 0.05 was considered to be statistically significant.

## Results

### miRNA Screening and Validation

**FIGURE 1:**
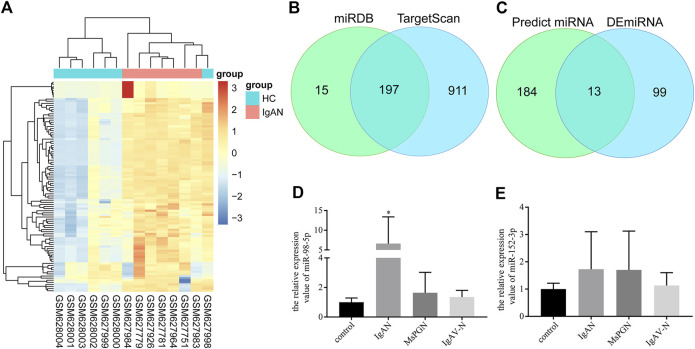
miRNA screening and validation. **(A)** Heatmap of DEmiRNAs in GSE25590. Red and blue: upregulated and downregulated. Columns represent different samples, rows indicates different genes. Significantly differential miRNA were defined by |log_2_(FC)|>1 and *p*-value < 0.05. **(B)** Venn diagram showing the intersection miRNAs predicted by TargetScan and miRDB. **(C)** Venn diagram showing the intersection of DEmiRNAs and predicted miRNAs. **(D)** Validation of the differential expression of miR-98-5p in PBMCs among patients with IgAN, healthy controls, patients with MsPGN, and patients with IgAV-N (the fold change of IgAN vs. healthy control, MsPGN and IgAV-N were 6.60 ± 6.20 vs. 1.00 ± 0.26, 6.60 ± 6.20 vs. 1.64 ± 1.26 and 6.60 ± 6.20 vs. 1.35 ± 0.42, respectively, all *p* < 0.05). **(E)** Validation of the differential expression of miR-152-3p among different groups. Each bar represents the mean ± SD of six cases in each group. Compared with the other groups, *p < 0.05. Note: DEmiRNA, differentially expressed miRNAs; FC, fold change; PBMCs, peripheral blood mononuclear cells; IgAN, IgA nephropathy; MsPGN, mesangial proliferative glomerulonephritis; IgAV-N, immunoglobulin A vasculitis nephritis; SD, standard deviation.

First, 112 mature differentially expressed miRNAs were identified from the GSE25590 dataset, including 91 upregulated and 21 downregulated miRNAs (Supplementary Table S2), the DEmiRNAs heatmap were shown in Figure 1A. TargetScan and miRDB were then used to predict potential upstream regulated miRNAs of C1GALT1, and got 197 predicted miRNAs (Figure 1B, Supplementary Table S3). A total of 13 target miRNAs were then identified based on the intersection of DEmiRNAs and predicted miRNAs (Figure 1C, Supplementary Table S4). Then we selected miR-98-5p and miR-152-3p for further validation.

A total of 24 pediatric cases were enrolled in our study. The demographic and clinical features of these cases are shown in Table 1. The levels of miR-98-5p were significantly higher in patients with IgAN than those in healthy controls (IgAN vs. control: 6.60 ± 6.20 vs. 1.00 ± 0.26, *p* < 0.05). Then, we investigated whether miR-98-5p was upregulated only in IgAN. We compared the same miR-98-5p levels of patients with IgAN with patients diagnosed with MsPGN and IgAV-N and found that miR-98-5p levels were also upregulated in patients with IgAN (IgAN vs. MsPGN and IgAV-N: 6.60 ± 6.20 vs. 1.64 ± 1.26 and 1.35 ± 0.42 respectively, *p* < 0.05) (Figure 1D). These results confirmed that the upregulation of miR-98-5p expression was specific for IgAN. There were no significant differences in the levels of miR-152-3p when compared between groups (Figure 1E).

**TABLE 1:** Demographic and clinical features of the healthy control group, and patients with IgAN, MsPGN, and IgAV-N.

variables^a^	Healthy control (n = 6)	IgAN (n = 6)	MsPGN (n = 6)	IgAV-N (n = 6)	*p*
Age (years)	9.43 ± 2.85	10.60 ± 3.80	6.12 ± 5.43	9.72 ± 1.11	0.191
Male (%)	3 (50.0)	3 (50.0)	3 (50.0)	5 (83.3)	0.621^b^
Systolic BP (mmHg)	101.17 ± 7.78	102.50 ± 9.23	96.33 ± 7.99	103.17 ± 6.37	0.452
Diastolic BP (mmHg)	68.00 ± 2.45	63.33 ± 10.86	57.33 ± 8.94	66.83 ± 6.18	0.111
Serum albumin (g/L)	40.9 (40.2–42.38)	34.9 (26.7–38.7)	42.2 (31.5–43.9)	41.9 (35.5–43.6)	0.288
eGFR (ml/min/1.73 m^2^)	149.7 ± 19.1	164.2 ± 26.7	156.9 ± 31.8	179.6 ± 14.6	0.201
24 h urinary protein (mg)	—	905.7 (94.5–1,618.0)	429.2 (99.9–2,166.6)	145.7 (78.8–453.9)	0.002^c^

^a^ Normally distributed continuous variables (values were expressed as mean ± SD). Non-normally distributed continuous variables were expressed as the median (first quartile and third quartile). For qualitative variables, values are expressed as n (%).

^b^ Fisher’s exact chi-square test, χ^2^ = 2.161.

^c^ The pairwise comparison of IgAN, MsPGN and IgAV-N group showed no statistical significance.

Abbreviations: HC, Healthy Control; IgAN, IgA nephropathy; MsPGN, mesangial proliferative glomerulonephritis; IgAV-N, immunoglobulin A vasculitis nephritis (IgAV-N); SD, standard deviation; BP, blood presure.

### The Expression Levels of miR-98-5p, C1Galactosyltransferase1, and Galactose-Deficient IgA1, in IgA Nephropathy, and the Relationships Between These Different Factors

**FIGURE 2:**
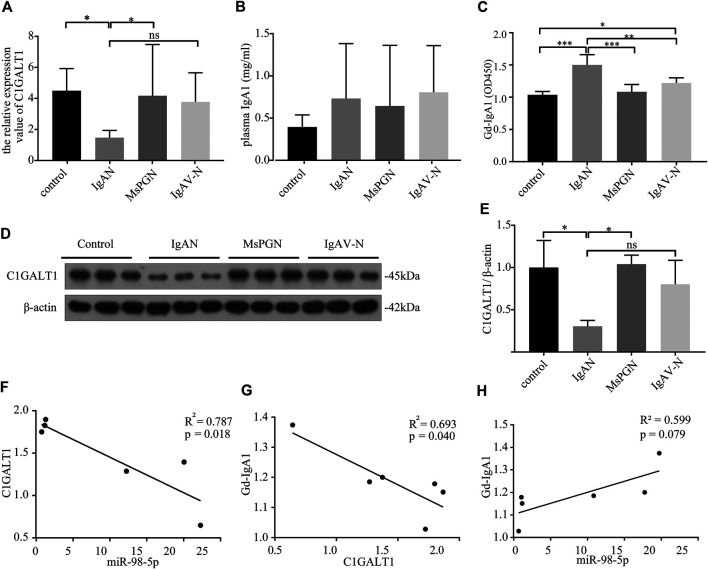
The expression levels and relationships among miR-98-5p, C1GALT1, and Gd-IgA1 in IgAN. **(A)** The expression levels of C1GALT1 mRNA were analyzed by qRT-PCR. The expression levels in control, IgAN, MsPGN and IgAV-N groups were 4.50 ± 1.30, 1.47 ± 0.43, 4.17 ± 3.01 and 3.76 ± 1.71 respectively. **(B)** The plasma IgA1 level was analyzed by ELISA, There were no significance between IgAN and other groups. **(C)** The relative level of Gd-IgA1 in the plasma was analyzed by VVA. The relative level of Gd-IgA1 in control, IgAN, MsPGN and IgAV-N groups were 1.04 ± 0.05, 1.50 ± 0.15, 1.08 ± 0.10 and 1.22 ± 0.07 respectively. **(D)** The expression of C1GALT1 protein was analyzed by Western blotting. **(E)** Fold changes were calculated by determining the ratios of the C1GALT1/β-actin band intensities. The semiquantitative relative level of C1GALT1 protein in control, IgAN, MsPGN and IgAV-N groups were 1.00 ± 0.26, 0.30 ± 0.06, 1.04 ± 0.09 and 0.80 ± 0.23. **(F)** The relationship between C1GALT1 and miR-98-5p. **(G)** The relationship between Gd-IgA1 and the mRNA of C1GALT1 **(H)** The relationship between Gd-IgA1 and miR-98-5p. Each bar represents the mean ± SD of six cases in each group. ns, no significance; *p < 0.05, **p < 0.01, ***p < 0.001. Note: C1GALT1, β-1, 3-galactosyltransferase; Gd-IgA1, galactose-deficient IgA1; IgAN, IgA nephropathy; qRT-PCR, quantitative real-time polymerase chain reaction; ELISA, enzyme-linked immunosorbent assay; VVA, Vicia Villosa lectin-binding assay; SD, standard deviation.

C1GALT1 is a necessary enzyme for the addition of galactose in the process of O-glycosylation and is directly associated with the onset of IgAN. We tested the mRNA expression levels of C1GALT1 in PBMCs by qRT-PCR and found a significant reduction in patients with IgAN when compared to healthy controls and patients with MsPGN (IgAN vs. healthy control and MsPGN: 1.47 ± 0.43 vs. 4.50 ± 1.30 and 4.17 ± 3.01, *p* < 0.05). There was no significant difference between patients with IgAV-N and IgAN (Figure 2A). We also measured the protein levels of C1GALT1 in PBMCs and found that the results were consistent with mRNA levels (Figures 2D,E). We then measured the plasma IgA1 concentration in the four groups and found no significant difference when compared between the different groups (Figure 2B). Next, we evaluated the levels of Gd-IgA1 in plasma samples from the same cases by VVA. Analysis showed that the levels of Gd-IgA1 were significantly higher in patients with IgAN than healthy controls (IgAN vs. control: 1.50 ± 0.15 vs. 1.04 ± 0.05, *p* < 0.001), patients with MsPGN (IgAN vs. MsPGN: 1.50 ± 0.15 vs. 1.08 ± 0.10, *p* < 0.001), and patients with IgAV-N (IgAN vs. IgAV-N: 1.50 ± 0.15 vs. 1.22 ± 0.07, *p* < 0.01). Furthermore, we found that the levels of Gd-IgA1 were significantly higher in patients with IgAV-N than in healthy controls (IgAV-N vs. control: 1.22 ± 0.07 vs. 1.04 ± 0.05, *p* < 0.05) (Figure 2C). These results suggest that C1GALT1 was significantly downregulated in IgAN and IgAV-N, but more significantly in IgAN; Gd-IgA1 was significantly upregulated in IgAN and IgAV-N, but more significantly in IgAN. Both C1GALT1 and Gd-IgA1 abnormal involved in IgAN pathogenesis.

Pearson correlation analysis showed that the expression levels of miR-98-5p and C1GALT1 mRNA were negatively correlated (R^2^ = 0.787, *p* = 0.018; Figure 2F); the C1GALT1 mRNA expression and Gd-IgA1 levels were negatively correlated (R^2^ = 0.793, *p* = 0.040; Figure 2G); and the expression of miR-98-5p and the levels of Gd-IgA1 were not significantly correlated (R^2^ = 0.599, *p* = 0.079; Figure 2H). These results suggest that C1GALT1 might be a target gene of miR-98-5p.

### miR-98-5p Regulates C1Galactosyltransferase1 Expression and Galactose-Deficient IgA1 Levels in DAKIKI Cells

PBMCs include lymphocytes (including both T and B lymphocytes) and monocytes. B lymphocytes can secrete antibodies in response to antigen stimulation. IgA is the most secreted antibody (Mestecky et al., 1986), and only IgA and IgD contain O-glycosylation in immunoglobulins (Smith et al., 2006). O-glycosylation requires C1GALT1. The O-glycosylation of IgD is normal in IgAN (Smith et al., 2006), this demonstrates the fact that the most affected cells are those that are IgA1-positive.

Next, we carried out *in vitro* experiments using human B lymphoma DAKIKI cells which are known to produce IgA1. The expression of C1GALT1 was almost completely lost, at both the mRNA and protein levels, when cells were treated with si-C1GALT1 when compared with cells treated with si-Scramble (si-C1GALT1 vs. si-Scramble: mRNA: 0.24 ± 0.02 vs. 0.92 ± 0.06, *p* < 0.001; protein: 0.29 ± 0.12 vs. 1.02 ± 0.15, *p* < 0.05) (Supplementary Figure S1A–C). The levels of Gd-IgA1 in the supernatant of DAKIKI cells was significantly elevated in the si-C1GALT1-treated group (si-C1GALT1 vs .si-Scramble: 0.47 ± 0.01 vs. 0.32 ± 0.00, *p* < 0.001) (Supplementary Figure S1D). The expression of C1GALT1 was significantly increased, both at the mRNA and protein levels, in cells that had been treated with pcDNA-C1GALT1 when compared with those treated with the pcDNA-vector (pcDNA-C1GALT1 vs. pcDNA-vector: mRNA: 4.47 ± 0.13 vs. 0.96 ± 0.12, *p* < 0.001, protein: 1.95 ± 0.39 vs. 0.99 ± 0.24, *p* < 0.001) (Supplementary Figure S1E–G). The levels of Gd-IgA1 in the supernatant of DAKIKI cells was significantly reduced in the pcDNA-C1GALT1-treated group (pcDNA-C1GALT1 vs. pcDNA-vector: 0.21 ± 0.00 vs. 0.31 ± 0.01, *p* < 0.001) (Supplementary Figure S1H). These results indicated that C1GALT1 negatively regulated the production of Gd-IgA1.

**FIGURE 3:**
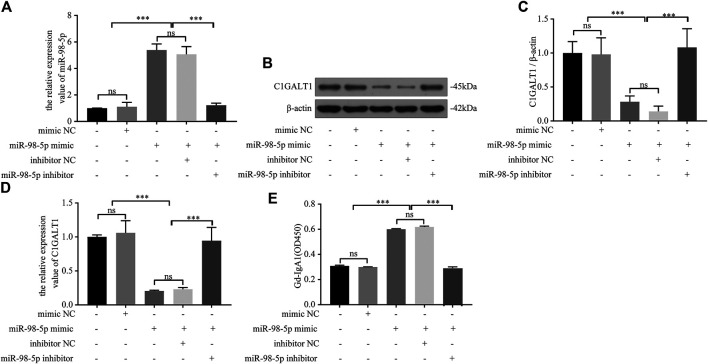
miR-98-5p regulated the expression of C1GALT1 and Gd-IgA1 levels in DAKIKI cells. **(A)** The expression level of miR-98-5p was analyzed by qRT-PCR in DAKIKI cells. The relative expression level of miR-98-5p in the blank, mimic NC, miR-98-5p mimic, miR-98-5p mimic + inhibitor NC, and miR-98-5p mimic + miR-98-5p inhibitor groups were 1.00 ± 0.01, 1.11 ± 0.27, 5.39 ± 0.38, 5.07 ± 0.50 and 1.22 ± 0.13. **(B)** The expression of C1GALT1 protein was analyzed by western blotting in DAKIKI cells. **(C)** Fold changes were calculated by determining the ratios of C1GALT1/β-actin band intensities in DAKIKI cells. The semiquantitative relative level of C1GALT1 protein in the blank, mimic NC, miR-98-5p mimic, miR-98-5p mimic + inhibitor NC, and miR-98-5p mimic + miR-98-5p inhibitor groups were 1.00 ± 0.14, 0.98 ± 0.20, 0.28 ± 0.07, 014 ± 0.06 and 1.08 ± 0.22. **(D)** The expression levels of C1GALT1 mRNA were analyzed by qRT-PCR in DAKIKI cells. The relative expression levels of C1GALT1 mRNA in the blank, mimic NC, miR-98-5p mimic, miR-98-5p mimic + inhibitor NC, and miR-98-5p mimic + miR-98-5p inhibitor groups were 1.00 ± 0.02, 1.06 ± 0.15, 0.21 ± 0.01, 0.23 ± 0.02 and 0.95 ± 0.16. **(E)** The relative levels of Gd-IgA1 level were analyzed by VVA in the supernatant of DAKIKI cells. The relative levels of Gd-IgA1 level in the blank, mimic NC, miR-98-5p mimic, miR-98-5p mimic + inhibitor NC, and miR-98-5p mimic + miR-98-5p inhibitor groups were 0.31 ± 0.00, 0.30 ± 0.00, 0.60 ± 0.00, 0.62 ± 0.01 and 0.29 ± 0.01. ns, no significance; ***p < 0.001. Note: C1GALT1, β-1, 3-galactosyltransferase; Gd-IgA1, galactose-deficient IgA1; qRT-PCR, quantitative real-time polymerase chain reaction; VVA, Vicia Villosa lectin-binding assay.

Next, we confirmed the causal relationship between miR-98-5p expression and Gd-IgA1 secretion by carrying out *in vitro* experiments. We transfected DAKIKI cells with blank, mimic NC, miR-98-5p mimic, miR-98-5p mimic + inhibitor NC, and miR-98-5p mimic + miR-98-5p inhibitor. First, we proved that the transfection had been successful by detecting the expression of miR-98-5p (miR-98-5p mimic vs. blank and mimic NC: 5.39 ± 0.38 vs. 1.00 ± 0.01and 1.11 ± 0.27, miR-98-5p mimic vs. miR-98-5p mimic + miR-98-5p inhibitor: 5.39 ± 0.38 vs. 1.22 ± 0.13, all *p* < 0.001) (Figure 3A). C1GALT1 protein and mRNA expression were significantly downregulated in the miR-98-5p mimic and miR-98-5p mimic + inhibitor NC groups (miR-98-5p mimic and miR-98-5p mimic + inhibitor NC vs. blank: 0.28 ± 0.07 and 0.14 ± 0.06 vs. 1.00 ± 0.14, *p* < 0.001). Co-transfection with the miR-98-5p inhibitor reversed this effect (Figures 3B–D). The levels of Gd-IgA1 in the supernatant were increased significantly in the miR-98-5p mimic and miR-98-5p mimic + inhibitor NC groups (miR-98-5p mimic and miR-98-5p mimic + inhibitor NC vs. blank and mimic NC: 0.60 ± 0.00 and 0.62 ± 0.01 vs. 0.31 ± 0.00 and 0.30 ± 0.00, *p* < 0.001); this effect was reversed by the miR-98-5p inhibitor (Figure 3E). These results indicated that miR-98-5p downregulated the expression of C1GALT1 and upregulated the expression of Gd-IgA1.

### Identification of miR-98-5p Targeting C1Galactosyltransferase1

**FIGURE 4:**
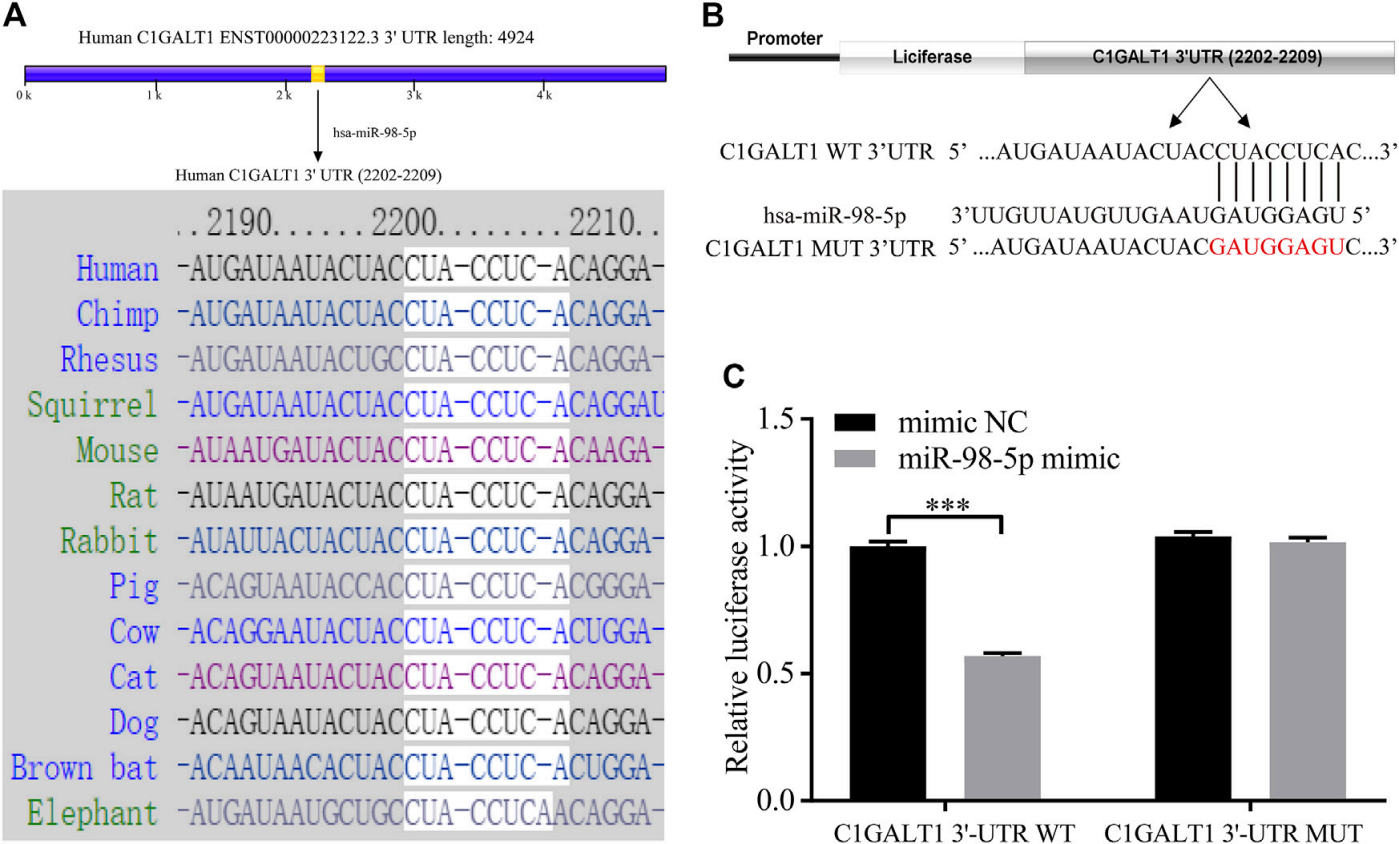
C1GALT1 represents a potential target gene of miR-98-5p. **(A)** Schematic diagram depicting the prediction of C1GALT1 as a potential target gene of miR-98-5p, as determined by TargetScan. **(B)** Schematic view of the luciferase reporter vector. **(C)** Relative luciferase activity of the C1GALT1-3′UTR WT or C1GALT1-3’UTR MUT reporter co-transfected with miR-98-5p mimic or mimic NC. The relative luciferase activity of C1GALT1-3’UTR WT reporter in mimic NC and miR-98-5p were 1.00 ± 0.16 and 0.57 ± 0.01; the relative luciferase activity of C1GALT1-3’UTR MUT reporter in mimic NC and miR-98-5p were 1.04 ± 0.15 and 1.02 ± 0.02. NC, negative control. ***p < 0.001. Note: C1GALT1, β-1, 3-galactosyltransferase; WT, wild type; MUT, mutant type.

Next, we used TargetScan and miRDB to predict the upstream miRNAs that regulated C1GALT1 and found that miR-98-5p could target C1GALT1 with good levels of interspecies conservation (Figure 4A). Next, we confirmed this hypothesis by using a standard dual-luciferase reporter assay. We constructed and cloned wild-type and mutant-type C1GALT1-3’-UTR sequences (C1GALT1-WT and C1GALT1-MUT) into the pHG-MirTarget-C1GALT1 luciferase reporter vector (Figure 4B). Co-transfection with the C1GALT1-WT and miR-98-5p mimic significantly reduced the relative luciferase activity when compared to that in the NC mimic group (mimic NC vs. miR-98-5p mimic: 1.00 ± 0.16 vs. 0.57 ± 0.01, *p* < 0.001); co-transfection with the C1GALT1-MUT and miR-98-5p mimic or the NC mimic did not affect fluorescence intensity (Figure 4C). These results proved that miR-98-5p might specifically target C1GALT1.

### miR-98-5p Upregulated Galactose-Deficient IgA1 Levels by Targeting C1Galactosyltransferase1 in DAKIKI Cells

**FIGURE 5:**
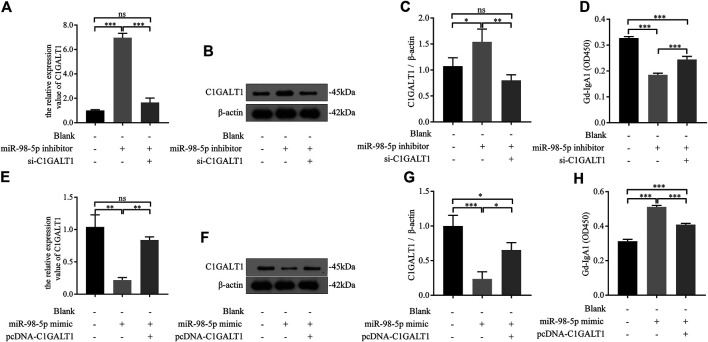
miR-98–5p upregulated the levels of Gd-IgA1 by targeting C1GALT1 in DAKIKI cells. **(A,E)** The expression levels of C1GALT1 mRNA were analyzed by qRT-PCR in DAKIKI cells. The relative expression level of C1GALT1 mRNA in the blank, miR-98-5p inhibitor and miR-98-5p inhibitor + si-C1GALT1 groups were 1.00 ± 0.05, 6.97 ± 0.29 and 1.66 ± 0.29. The relative expression level of C1GALT1 mRNA in the blank, miR-98-5p mimic and miR-98-5p mimic + pcDNA-C1GALT1 groups were 1.04 ± 0.15, 0.22 ± 0.03 and 0.84 ± 0.04. **(B,F)** The expression of C1GALT1 protein were analyzed by Western blotting in DAKIKI cells. **(C,G)** Fold changes were calculated by determining the ratios of the C1GALT1/β-actin band intensities in DAKIKI cells. The semiquantitative relative level of C1GALT1 protein in the blank, miR-98-5p inhibitor and miR-98-5p inhibitor + si-C1GALT1 groups were 1.07 ± 0.13, 1.54 ± 0.20 and 0.80 ± 0.09. The semiquantitative relative level of C1GALT1 protein in the blank, miR-98-5p mimic and miR-98-5p mimic + pcDNA-C1GALT1 groups were 1.00 ± 0.13, 0.24 ± 0.08 and 0.65 ± 0.09. **(D,H)** The relative levels of Gd-IgA1 were analyzed by VVA in the supernatant of DAKIKI cells. The relative levels of Gd-IgA1 in the blank, miR-98-5p inhibitor and miR-98-5p inhibitor + si-C1GALT1 groups were 0.33 ± 0.00, 0.19 ± 0.01 and 0.24 ± 0.01. The relative levels of Gd-IgA1 in the blank, miR-98-5p mimic and miR-98-5p mimic + pcDNA-C1GALT1 groups were 0.31 ± 0.01, 0.51 ± 0.01 and 0.41 ± 0.01. ns, no significance; *p < 0.05, **p < 0.01, ***p < 0.001. Note: Gd-IgA1, galactose-deficient IgA1; C1GALT1, β-1, 3-galactosyltransferase; qRT-PCR, quantitative real-time polymerase chain reaction; VVA, Vicia Villosa lectin-binding assay.

We transfected DAKIKI cells with miR-98-5p inhibitor, mimics, or co-transfected with si-C1GALT1 or pcDNA-C1GALT1 to confirm whether C1GALT1 was a functional target gene of miR-98-5p. Non-transfected cells served as a control group (blank group). The miR-98-5p inhibitor upregulated the mRNA and protein levels of C1GALT1 when compared with the blank group; this effect was significantly reversed by si-C1GALT1 (miR-98-5p inhibitor vs. blank and miR-98-5p inhibitor + si-C1GALT1: mRNA: 6.97 ± 0.29 vs. 1.00 ± 0.05 and 1.66 ± 0.29, both *p* < 0.001; protein: 1.54 ± 0.20 vs. 1.07 ± 0.13 and 0.80 ± 0.09, *p* < 0.05 and *p* < 0.01) (Figures 5A–C). When compared with the blank group, the downregulation of miR-98-5p significantly inhibited Gd-IgA secretion; this effect was significantly rescued by si-C1GALT1 (miR-98-5p inhibitor vs. blank and miR-98–5p inhibitor + si-C1GALT1: 0.19 ± 0.01 vs. 0.33 ± 0.00 and 0.19 ± 0.01, both *p* < 0.001) (Figure 5D). When compared with the blank group, the miR-98-5p mimic notably significantly downregulated the mRNA and protein levels of C1GALT1; this effect was reversed by pcDNA-C1GALT1 (miR-98-5p mimic vs. blank and miR-98-5p mimic + pcDNA-C1GALT1: mRNA: 0.22 ± 0.03 vs. 1.04 ± 0.15 and 0.84 ± 0.04, both *p* < 0.01; protein: 0.24 ± 0.08 vs. 1.00 ± 0.13 and 0.65 ± 0.09, *p* < 0.001 and *p* < 0.05) (Figures 5E–G). When compared with the blank group, the overexpression of miR-98-5p significantly promoted Gd-IgA secretion; this effect was significantly reversed by pcDNA-C1GALT1 (miR-98-5p mimic vs. blank and miR-98-5p mimic + pcDNA-C1GALT1: 0.51 ± 0.01 vs. 0.31 ± 0.01 and 0.41 ± 0.01, both *p* < 0.001) (Figure 5H).

### Astragaloside IV Downregulated Galactose-Deficient IgA1 Levels *via* miR-98-5p in DAKIKI Cells

AS-IV has multiple pharmacological effects, including antioxidant and anti-inflammatory effects and cardiovascular and renal protection. Combined with our previous findings that miR-98-5p promoted the secretion of Gd-IgA1, we hypothesized that AS-IV might interact with miR-98-5p to function as a renal protector.

**FIGURE 6:**
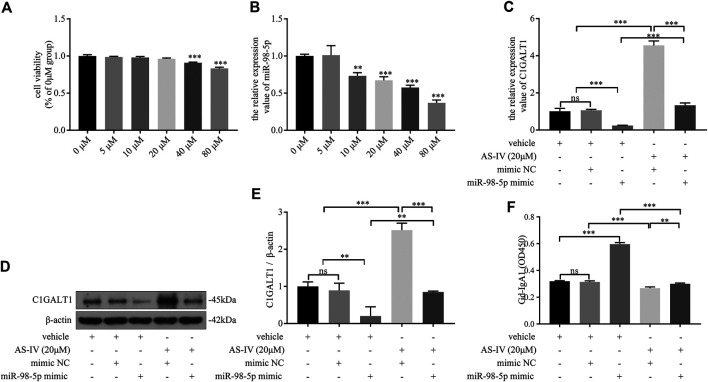
AS-IV downregulated Gd-IgA1 levels *via* miR-98-5p in DAKIKI cells. **(A)** Different concentrations of AS-IV toxicity were determined by CCK-8 assay. The cell viability (of the percentage of 0 μM in) in 5, 10, 20, 40 and 80 μM groups were 0.99 ± 0.01, 0.98 ± 0.01, 0.96 ± 0.01, 0.91 ± 0.01, 0.83 ± 0.1. **(B)** The expression levels of miR-98-5p were analyzed by qRT-PCR in DAKIKI cells treated with different concentrations of AS-IV. The relative expression level of miR-98-5p in the 0, 5, 10, 20, 40 and 80 μM groups were 1.00 ± 0.02, 1.01 ± 0.10, 0.73 ± 0.04, 0.67 ± 0.04, 0.58 ± 0.03 and 0.37 ± 0.03. **(C)** The expression levels of C1GALT1 mRNA were analyzed by qRT-PCR in DAKIKI cells. The relative expression levels of C1GALT1 mRNA in the vehicle, vehicle + mimic NC, vehicle + miR-98-5p, AS-IV + mimic NC and AS-IV + miR-98-5p groups were 1.01 ± 0.13, 1.06 ± 0.04, 0.24 ± 0.02, 4.56 ± 0.20 and 1.33 ± 0.10. **(D)** The expression of C1GALT1 protein were analyzed by western blotting in DAKIKI cells. **(E)** Fold changes were calculated by determining the ratios of the C1GALT1/β-actin band intensities in DAKIKI cells. The semiquantitative relative level of C1GALT1 protein in the vehicle, vehicle + mimic NC, vehicle + miR-98-5p, AS-IV + mimic NC and AS-IV + miR-98-5p groups were 1.00 ± 0.10, 0.89 ± 0.16, 0.20 ± 0.20, 2.52 ± 0.15, 0.85 ± 0.02, and 0.37 ± 0.03. **(F)** The relative levels of Gd-IgA1 were analyzed by VVA in the supernatant of DAKIKI cells. The relative levels of Gd-IgA1 in the vehicle, vehicle + mimic NC, vehicle + miR-98-5p, AS-IV + mimic NC and AS-IV + miR-98-5p groups were 0.32 ± 0.00, 0.31 ± 0.01, 0.60 ± 0.01, 0.27 ± 0.01, and 0.30 ± 0.01. ns, no significance; **p < 0.01, ***p < 0.001. Note: AS-IV, Astragaloside IV; Gd-IgA1, galactose-deficient IgA1; CCK-8, cell counting kit-8 assay; qRT-PCR, quantitative real-time polymerase chain reaction; C1GALT1, β-1, 3-galactosyltransferase; VVA, Vicia Villosa lectin-binding assay.

First, DAKIKI cells were treated with different concentrations of AS-IV for 24 h. The toxicity of AS-IV was then tested by CCK-8 assay. The cell viability began to decline when the concentration of AS-IV exceeded 20 μm/ml (Figure 6A). As the concentration of AS-IV increased, the levels of miR-98-5p decreased (Figure 6B). These results indicated that AS-IV inhibited the expression of miR-98-5p in DAKIKI cells. Based on the toxicity of AS-IV, and the levels of miR-98-5p, we chose 20 μm/ml of AS-IV to perform further experiments. The mRNA and protein levels of C1GALT1 were significantly downregulated in the miR-98-5p mimic group when compared to the vehicle and mimic NC group, but was reversed by AS-IV treatment (miR-98-5p mimic + vehicle vs.: miR-98-5p mimic + AS-IV: mRNA: 0.24 ± 0.02 vs. 1.33 ± 0.10, *p* < 0.001; protein: 0.20 ± 0.20 vs. 0.37 ± 0.03, *p* < 0.01) (Figures 6C–E). In contrast to C1GALT1, the levels of Gd-IgA1 were significantly higher in the miR-98–5p mimic group than the vehicle and the mimic NC group (miR-98–5p mimic + vehicle vs. vehicle and vehicle + mimic NC: 4.57 ± 0.20 vs. 1.01 ± 0.13 and 1.06 ± 0.04, both *p* < 0.001), but could also be reversed by AS-IV treatment (miR-98-5p mimic + vehicle vs.: miR-98-5p mimic + AS-IV: 0.60 ± 0.01 vs. 0.30 ± 0.01, *p* < 0.001) (Figure 6F). Collectively, these data showed that AS-IV downregulated the levels of Gd-IgA1 by downregulating miR-98-5p levels in DAKIKI cells.

## Discussion

The first and most important factor underlying IgAN is abnormalities in IgA itself. The diagnosis of IgAN is based on certain pathological features: IgA-containing CIC deposition, usually with IgG and C3 co-deposition (Irabu et al., 2020). The form of IgA that is deposited in renal tissues is mainly Gd-IgA1 with deficient Gal residues on the O-glycans in the hinge region of the heavy chains. At present, IgAN is regarded as an autoimmune disease; the pathogenesis of this condition is described by the “multi-hit hypothesis” (Moroni et al., 2019). Based on the multi-hit hypothesis, Gd-IgA1 is the origin and driving factor of IgAN.

IgA1 is specific for humans and hominoid primates. IgA1 contains a core of one O-glycans composed of GalNAc with β1,3-linked Gal (Lai et al., 2019). C1GALT1 catalyzes the transfer of Gal from UDP-Gal to GalNAc-alpha-1-Ser/Thr to generate the common core one O-glycan structure. A reduction in the activity or expression levels of C1GALT1 can lead to the abnormal addition of Gal residues, thus resulting in the formation of Gd-IgA1. Numerous studies have confirmed that Gd-IgA1 is contained within the CIC and that deposition in the mesangial tissues of patients with IgAN was mainly Gd-IgA1 (Maixnerova et al., 2019). We found that the levels of Gd-IgA1 increased in the plasma, and that the mRNA and protein levels of C1GALT1 decreased in the PBMCs of patients with IgAN. The levels of C1GALT1 were negatively correlated with the levels of Gd-IgA1 in DAKIKI cells. This is consistent with previous literature reports (Liu et al., 2020).

Previous studies have used Genome-Wide Association Studies (GWAS) to investigate whether C1GALT1 is genetically altered in the IgAN population. One study found that the variance in serum Gd-IgA1 levels in 7% of Europeans and 2% of East Asians could be explained by the C1GALT1 and C1GALTC1 loci (Kiryluk et al., 2017). This indicated that the C1GALT1 gene itself could only explain a small part of the observed variance of serum Gd-IgA1 in patients with IgAN. This indicates that further research is needed with regards to the regulatory network in IgAN. Studies have demonstrated that C1GALT1 can be regulated by cytokines, Golgi matrix protein 130 (GM130), as well as by miRNA (Xiao et al., 2017; Selvaskandan et al., 2018; Wang C. et al., 2019).

miRNAs regulate target mRNAs at the post-transcriptional level by inducing mRNA degradation or by inhibiting translation (Zhao et al., 2019). Therefore, the biological role of miRNA can be reflected by its regulated target gene. Numerous studies have found that miRNAs play a vital role in the occurrence and development of IgAN and can be used as non-invasive biomarkers for diagnosis and or the evaluation of renal damage (Selvaskandan et al., 2018). Recent insights have also revealed that miRNAs such as miR-148b, let-7b, and miR-155, play crucial roles in IgAN (Wang et al., 2011; Serino et al., 2012; Serino et al., 2015).

By analyzing the GSE25590 miRNA microarray, we identified 112 mature DEmiRNAs, including 91 upregulated and 21 downregulated miRNAs. Next, we set C1GALT1 as the target gene and used Targetscan and miRDB to predict 197 target miRNAs. Next, we identified the intersection of the DEmiRNAs and predicted miRNAs and identified a total of 13 target miRNAs for further study. The expression trend for the miRNAs was opposite to that for the target gene; consequently, we excluded the down-regulated hsa-miR-590-3p and hsa-miR-488-3p. We set the filter conditions of the Pct value in TargetScan at > 0.75, and the TargetScore in miRDB >60, to compare conservation between species and the possibility of miRNA targeting target genes; this practice led to the exclusion of hsa-miR-488-3p and hsa-miR-543. Previous studies have demonstrated that the increased expression of miR-148 and let-7b promotes Gd-IgA1 secretion by targeting C1GALT1 and UDP-N-acetyl-α-d-galactosamine:polypeptide N-acetylgalactosaminyltransferase 2 (GALNT2), respectively (Serino et al., 2012; Serino et al., 2015). Therefore, we also excluded miR-148b-3p. Since let-7s exhibit a similar structure and function, we also excluded hsa-let-7a-5p, hsa-let-7b-5p hsa-let-7c-5p, hsa-let-7d-5p, hsa-let-7f-5p, hsa-let-7g-5p, and hsa-let-7i-5p. Previous reports have revealed that miR-98 (now referred to as miR-98-5p) is associated with systemic lupus erythematosus, diabetic nephropathy, and B cell-related immune inflammations (Gonzalez-Martin et al., 2016). However, whether this plays a role in IgAN remains unknown. Therefore, we selected miR-98-5p and miR-152-3p for further validation. PCR results showed that only miR-98-5p was upregulated in pediatric patients of IgAN and was therefore selected for further analysis. miR-98-5p is highly conserved in terms of sequence and spatiotemporal expression across different species and exhibits a range of physiological functions, including the inhibition of cytokine synthesis, the regulation of lipid metabolism, and regulation of the immune system (Chen et al., 2017; Sun et al., 2018). Since Gd-IgA1 is synthesized by B cells, any form of B cell dysfunction may promote its secretion.

We found that the levels of miR-98-5p were negatively correlated with C1GALT1 in PBMCs from pediatric patients with IgAN and in DAKIKI cells. miR-98-5p significantly inhibited the activity of the wild-type C1GALT1 gene; the mutant-type was not affected, as confirmed by dual-luciferase reporter gene experiments. We then conducted a rescue experiment to verify the relationship between miR-98-5p and C1GALT1. Our experiments found that the increased levels of Gd-IgA1 induced by the miR-98-5p mimic could be significantly reversed by the overexpression of C1GAL1. Furthermore, the reduction of Gd-IgA1 secretion caused by the miR-98-5p inhibitor could be impaired by the silencing of C1GALT1. These results demonstrated that the stimulatory effect of miR-98-5p on Gd-IgA1 secretion was mediated by the downregulation of C1GALT1.

We measured the mRNA and protein levels of C1GALT1 in healthy controls and pediatric patients with MsPGN, IgAV-N, and IgAN, and found the levels of C1GALT1 were significantly reduced in patients with IgAN and IgAV-N. We therefore hypothesize that the occurrence and development of IgAN and IgAV-N may both be related to Gd-IgA1. However, miR-98-5p was only significantly increased in patients with IgAN, thus suggesting that the regulatory mechanism of C1GALT1 in IgAN and IgAV-N was different. However, our sample size was small; larger sample sizes are now needed to verify our current findings.

*Astragalus membranaceus* (Huangqi in Chinese) is a traditional Chinese herbal medicine that is commonly used for clinical treatments. AS-IV is an active ingredient of *Astragalus membranaceus* and has been shown to exert cardioprotective, neuroprotective, and renoprotective activities (Wang E. et al., 2020; Wang Z. et al., 2020; Xia et al., 2020). Thus, we hypothesized that AS-IV might also have a protective effect on IgAN. In our investigations, we treated DAKIKI cells with AS-IV and found that an elevated concentration of AS-IV could down-regulate miR-98-5p. Next, we further investigated the relationship between AS-IV and miR-98-5p in DAKIKI cells. Data revealed that 20 μM/mL of AS-IV could abolish the regulatory effect caused by the overexpression of miR-98-5p on both C1GALT1 and Gd-IgA1, thus confirming that miR-98-5p might be a downstream molecule of AS-IV that regulates the levels of C1GALT1 and Gd-IgA1.

Our study has several limitations that need to be considered. First of all, the sample size was small; larger sample sizes are now needed to verify our findings. Secondly, the experiments were performed only with PBMCs and the DAKIKI cell line; we did not carry out any experiments in animal models because we do not have the experimental conditions to create humanized mice. Thirdly, this study only observed the effect of AS-IV on the DAKIKI cell line, not PBMCs from patients with IgAN. Although *Astragalus membranaceus* has been widely used for clinical treatment in China, AS-IV has not.

In conclusion, our results reveal that AS-IV could inhibit Gd-IgA1 secretion *via* miR-98-5p. Increased levels of miR-98-5p in pediatric IgAN patients might affect the glycosylation of IgA1 by targeting C1GALT1, thus leading to the excessive synthesis of Gd-IgA1. In addition, our analyses suggest that the pathogenesis of IgAN may differ from that of IgAV-N. Collectively, these results provide significant insight into the pathogenesis of IgAN and identify a potential therapeutic target.

## Data Availability

The raw data supporting the conclusions of this article will be made available by the authors, without undue reservation.
