# Supplementary material for: Astragaloside IV Inhibits Galactose-Deficient IgA1 Secretion via miR-98-5p in Pediatric IgA Nephropathy
Source: Front Pharmacol. 2021 Apr 16;12:658236. doi: 10.3389/fphar.2021.658236 (PMC8085534; doi:10.3389/fphar.2021.658236)
Supplement: Supplementary file 1 [file datasheet1.zip › Supplementary Tables/Supplementary Table 1.DOCX]

**Supplementary Table 1** Quantitative PCR primer and probe sequences for miR-98-5p, miR-152-3p, C1GALT1 and β-actin

| miR-98-5p | Forward | 5′- GGGCGGTGAGGTAGTAAGTTG-3′ |
| --- | --- | --- |
|  | Reverse | universal |
| miR-152-3p | Forward | 5′-CCGTGCATGACAGAACTTGGAAA-3′ |
|  | Reverse | universal |
| C1GALT1 | Forward | 5′-ATACGACCCTGAAGAACCCAT-3′ |
|  | Reverse | 5′-CATCTCCCCAGTGCTAAGTCT-3′ |
| U6 | Forward | 5′-CTCGCTTCGGCAGCACA-3′ |
|  | Reverse | 5′- AACGCTTCACGAATTTGCGT-3′ |
| β-actin | Forward | 5′- ACCCTGAAGTACCCCATCGAG-3′ |
|  | Reverse | 5′- AGCACAGCCTGGATAGCAAC-3′ |
