# Supplementary figures and images for: Astragaloside IV Inhibits Galactose-Deficient IgA1 Secretion via miR-98-5p in Pediatric IgA Nephropathy
Source: Front Pharmacol. 2021 Apr 16;12:658236. doi: 10.3389/fphar.2021.658236 (PMC8085534; doi:10.3389/fphar.2021.658236)

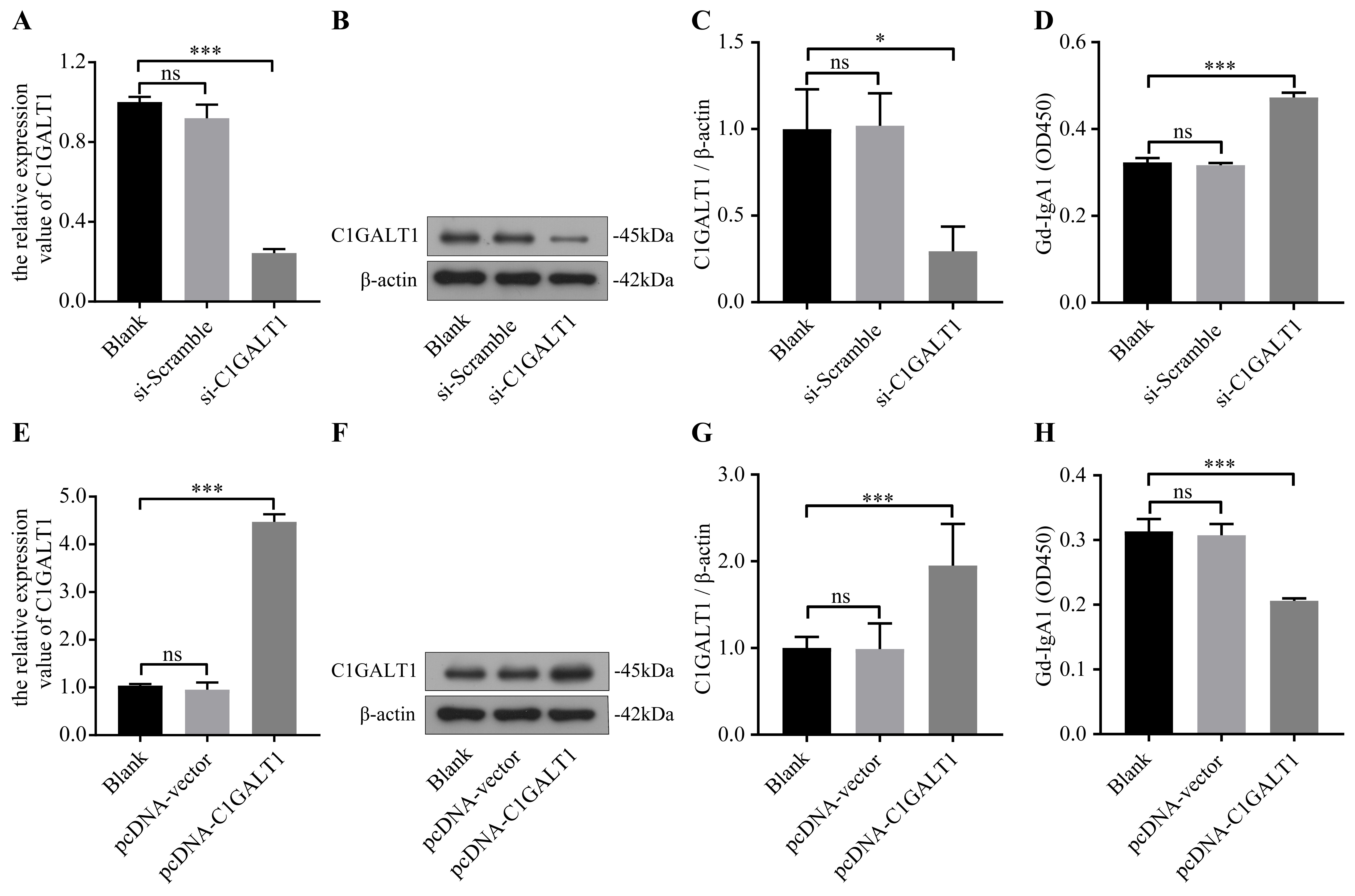

Supplement: Supplementary file 2 [file image1.tif]
